# A Case of Community-Acquired Tuberculosis in an Infant Presenting with Pneumonia Refractory to Antibiotic Therapy

**DOI:** 10.21980/J8X07M

**Published:** 2023-01-31

**Authors:** Audra N Iness, Andrea T Cruz, Scott R Dorfman, Esther M Sampayo

**Affiliations:** * Baylor College of Medicine, Department of Pediatrics, Houston, TX; ^ Baylor College of Medicine, Department of Pediatrics, Division of Pediatric Emergency Medicine, Houston, TX; † Baylor College of Medicine, Department of Pediatrics, Division of Pediatric Infectious Diseases, Houston, TX; ^^ Baylor College of Medicine and Texas Children’s Hospital, Edward B. Singleton Department of Pediatric Radiology, Houston, TX

## Abstract

Infant tuberculosis (TB) is a rare but potentially deadly infection and difficult to diagnose, especially in infants who may present with non-specific symptoms. Here, we report a case of an United States-born term infant with community-acquired miliary TB and no confirmed TB exposure history. The patient initially presented with respiratory distress at seven weeks of life with chest radiograph showing a right lower lobe (RLL) infiltrate. After failing multiple courses of treatment for community-acquired pneumonia and developing growth faltering, the patient had imaging findings suggestive of TB infection with CNS involvement. The diagnosis of TB was confirmed by QuantiFERON and purified protein derivative (PPD). In infants who fail conventional treatment for bacterial pneumonia, the differential should be broadened to consider alternative etiologies. Additionally, brain imaging should be performed in cases of disseminated TB despite negative cerebrospinal fluid (CSF) studies since these patients are at high risk of central nervous system (CNS) involvement.

**Topics:**

Tuberculosis, pneumonia, pediatrics, growth faltering.

**Figure f1-jetem-8-1-v18:**
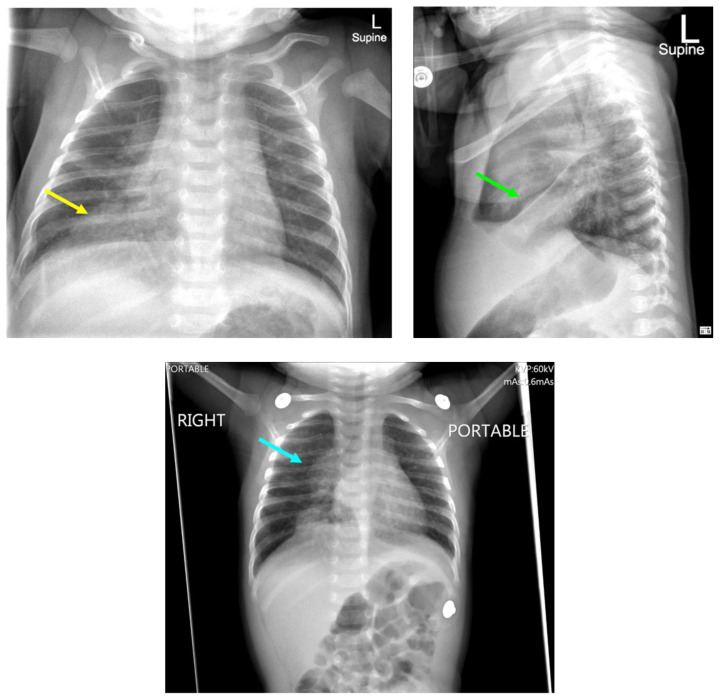


**Figure f2-jetem-8-1-v18:**
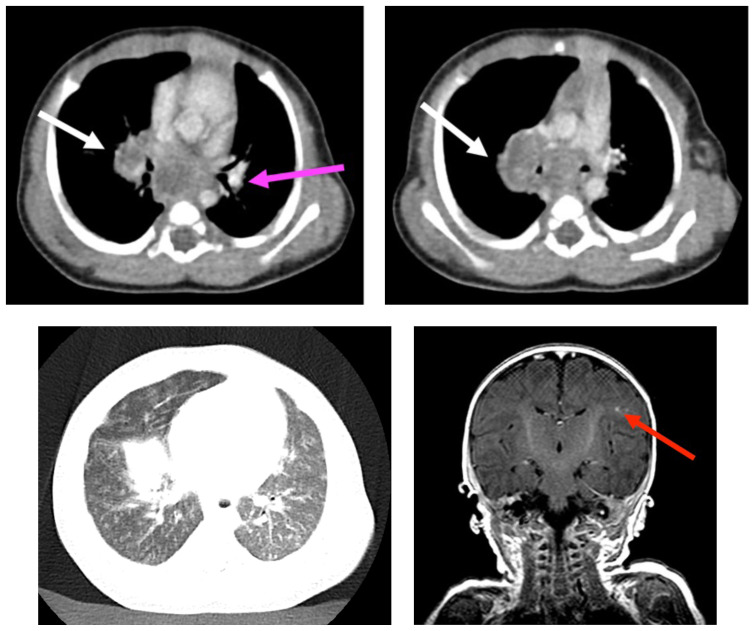


## Brief introduction

[Fig f1-jetem-8-1-v18][Fig f2-jetem-8-1-v18]Tuberculosis is a relatively rare infection in the United States with a rate of 2.2 per 100,000 persons. In 2020, there were 317 cases of TB among children less than 14 years of age, comprising 4% of all cases nationally.^1^ A systematic review and meta-analysis of U.S. cases from 1980–2016 reported a case fatality rate of 2% (N=12,741, 95% CI 0.5–7.4) for the 0–4-year-old age group.^2^ TB can present a diagnostic challenge in infants given the paucibacillary nature and relative lack of sensitivity of microbiologic detection. High clinical suspicion is required. Here, we discuss a case of an infant with disseminated TB and no confirmed risk factors.^3^

## Presenting concerns and clinical findings

A prior term male infant with an unremarkable neonatal course born to a mother with an uncomplicated pregnancy presented to the Emergency Department (ED) for the third time in 12 days. The patient presented with acute hypercarbic respiratory failure requiring noninvasive positive pressure ventilation, and with vomiting, diarrhea, and decreased oral intake at the 0.06th percentile on the growth curve.

## Significant findings

Chest radiographs during the initial presentation at seven weeks of life demonstrated right lower lobe (RLL) air space opacity on both PA and lateral views, compatible with pneumonia (referenced by yellow and green arrows, respectively). Repeat chest radiograph performed 12 days after the initial imaging revealed persistent right lower lobe opacity and right hilar fullness, seen as an opacified projection off of the mediastinal border as compared with the prior image, concerning for lymphadenopathy (designated by the aqua arrow). On the third presentation, computed tomography (CT) of the chest with intravenous contrast found persistent right lower lobe consolidation, innumerable 2–3 mm nodules, and surrounding ground glass opacities. This is best visualized as scattered areas of hyperdensity in the lung parenchyma. Axial images confirmed the presence of right hilar as well as subcarinal lymphadenopathy (indicated by white and pink arrows, respectively). Magnetic resonance imaging (MRI) of the brain with IV contrast was performed which showed a punctate focus of enhancement in the left precentral sulcus compatible with a tuberculoma (denoted with red arrow).

## Patient course

The patient initially presented at seven weeks of life for fever and respiratory distress with concern for right lower lobe pneumonia on chest radiograph. He received a full sepsis workup notable for leukocytosis with normal absolute neutrophil count (ANC), cerebrospinal fluid (CSF) with elevated WBC 32 (47% lymphs, 53% monos), normal glucose (62), elevated protein (57), as well as negative Gram stain and culture. The patient was discharged home with antibiotics for community-acquired pneumonia and re-admitted for similar concerns two days later. The chest radiograph was read as persistent right lower lobe opacity and lymphadenopathy. Urine studies and blood culture were again negative.

On the third ED presentation, the patient was admitted for respiratory failure and further workup of recurrent pneumonia. Chest computed tomography (CT) scan revealed multiple small bilateral pulmonary nodules, right hilar and subcarinal lymphadenopathy, consistent with a miliary pattern of disease. Empiric treatment was initiated for disseminated TB. A brain MRI was obtained to investigate the potential for CNS involvement and showed a focus of enhancement in the left precentral sulcus consistent with a small tuberculoma. Contemporaneous CSF studies were without pleocytosis; however, the patient was treated for intracranial disease based on imaging findings. Both the interferon gamma release assay and the TB skin test were positive. He was started on isoniazid, rifampin, levofloxacin, pyrazinamide, and ethionamide. Upon further history, the patient was born in the United States but there was no evidence of tuberculosis on chest imaging. By the end of therapy, he had radiographic resolution and was at the 40^th^ percentile for weight.

## Discussion

The differential diagnosis for refractory or recurrent pneumonia and growth faltering is broad, including primary pulmonary causes (cystic fibrosis, ciliary dyskinesia, congenital pulmonary airway malformation), malignancy, infectious etiology (tuberculosis, histoplasmosis, endocarditis with septic emboli), and primary immune deficiency. A high index of suspicion for tuberculosis is prudent in these cases with a thorough history warranted. Risk factors for tuberculosis infection include foreign-born or recent travel status or contact with persons at high risk of TB disease (persons in congregate settings, drug users, unprotected health care workers, and homeless persons). Vertical transmission from mother to neonate is also possible.^4^ Interestingly, a meta-analysis of studies from 1929–2015 found that <20% of TB transmission to children was attributable to household exposures, suggesting that community exposure may be more substantial than previously considered, consistent with the mode of acquisition in this case.^5^ Since pulmonary TB may be difficult to distinguish from community-acquired pneumonia, results of a recent retrospective cohort study in South Korea proposed that a delta neutrophil index (>1.0%) may be useful to rule out the possibility of pulmonary TB given its high negative predictive value, but its utility is dependent on regional prevalence.^6^

Importantly, infants are more likely to develop disseminated TB or TB meningitis than older individuals.^1^ Involvement of the CNS in TB infection develops in less than 2% of cases, half of which occur in children under age two. Chest radiography in infant TB may reveal miliary pattern (47%), multiple pulmonary nodules (11%), and lobar pneumonia (12%).^4^ Patients with a miliary pattern should be evaluated for meningitis and intracranial disease. Analysis of CSF usually demonstrates lymphocytosis, low glucose, and high protein concentrations, though CNS involvement with negative CSF studies was previously reported.^7^,^8^ In the present case, the patient had transient pleocytosis and elevated protein on CSF analysis, which interestingly temporally correlated with lobar pneumonia on imaging. Together, this further argues for CNS imaging studies as normal CSF parameters do not exclude intracranial involvement.

This case highlights the diagnostic dilemma associated with infant TB. Importantly, neuroimaging studies should be performed in infants with miliary disease and the inability of CSF findings to exclude intracranial involvement.

## Supplementary Information


























