# Supplementary figures and images for: A Case of Community-Acquired Tuberculosis in an Infant Presenting with Pneumonia Refractory to Antibiotic Therapy
Source: J Educ Teach Emerg Med. 2023 Jan 31;8(1):V18–22. doi: 10.21980/J8X07M (PMC10332766; doi:10.21980/J8X07M)

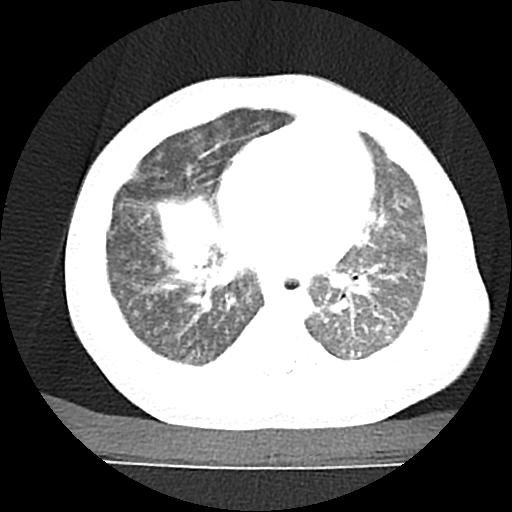

Supplement: Supplementary file 1 [file jetem-8-1-v18-supp1.jpg]

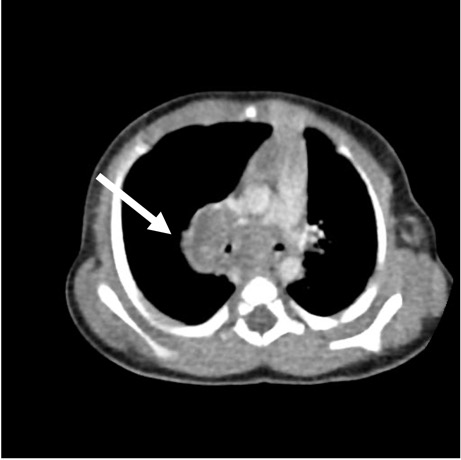

Supplement: Supplementary file 2 [file jetem-8-1-v18-supp2.jpg]

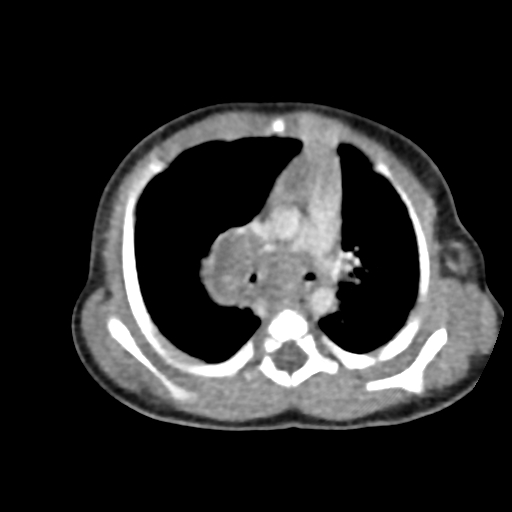

Supplement: Supplementary file 3 [file jetem-8-1-v18-supp3.jpg]

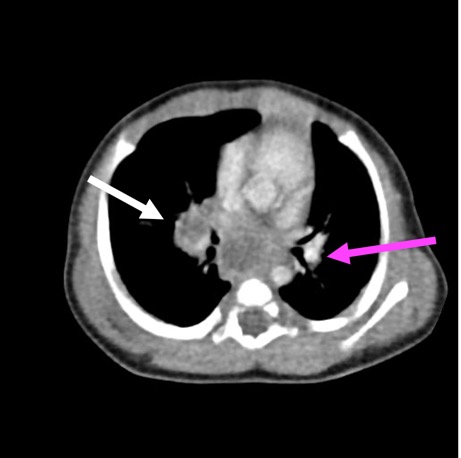

Supplement: Supplementary file 4 [file jetem-8-1-v18-supp4.jpg]

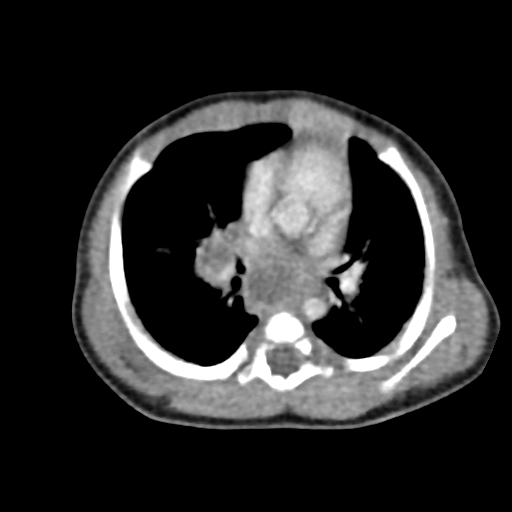

Supplement: Supplementary file 5 [file jetem-8-1-v18-supp5.jpg]

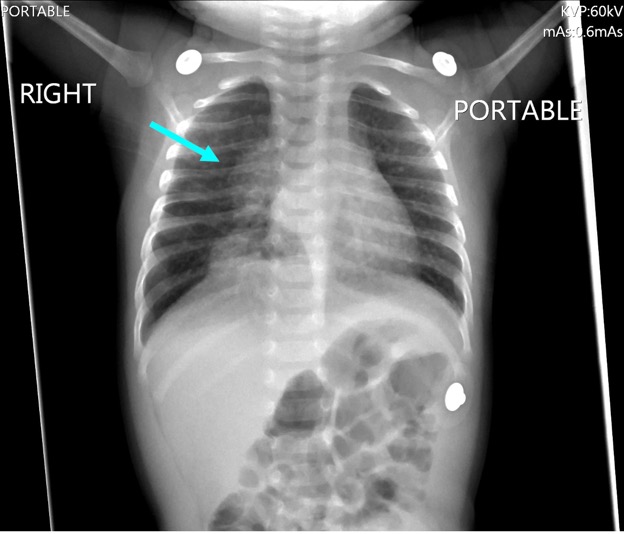

Supplement: Supplementary file 6 [file jetem-8-1-v18-supp6.jpg]

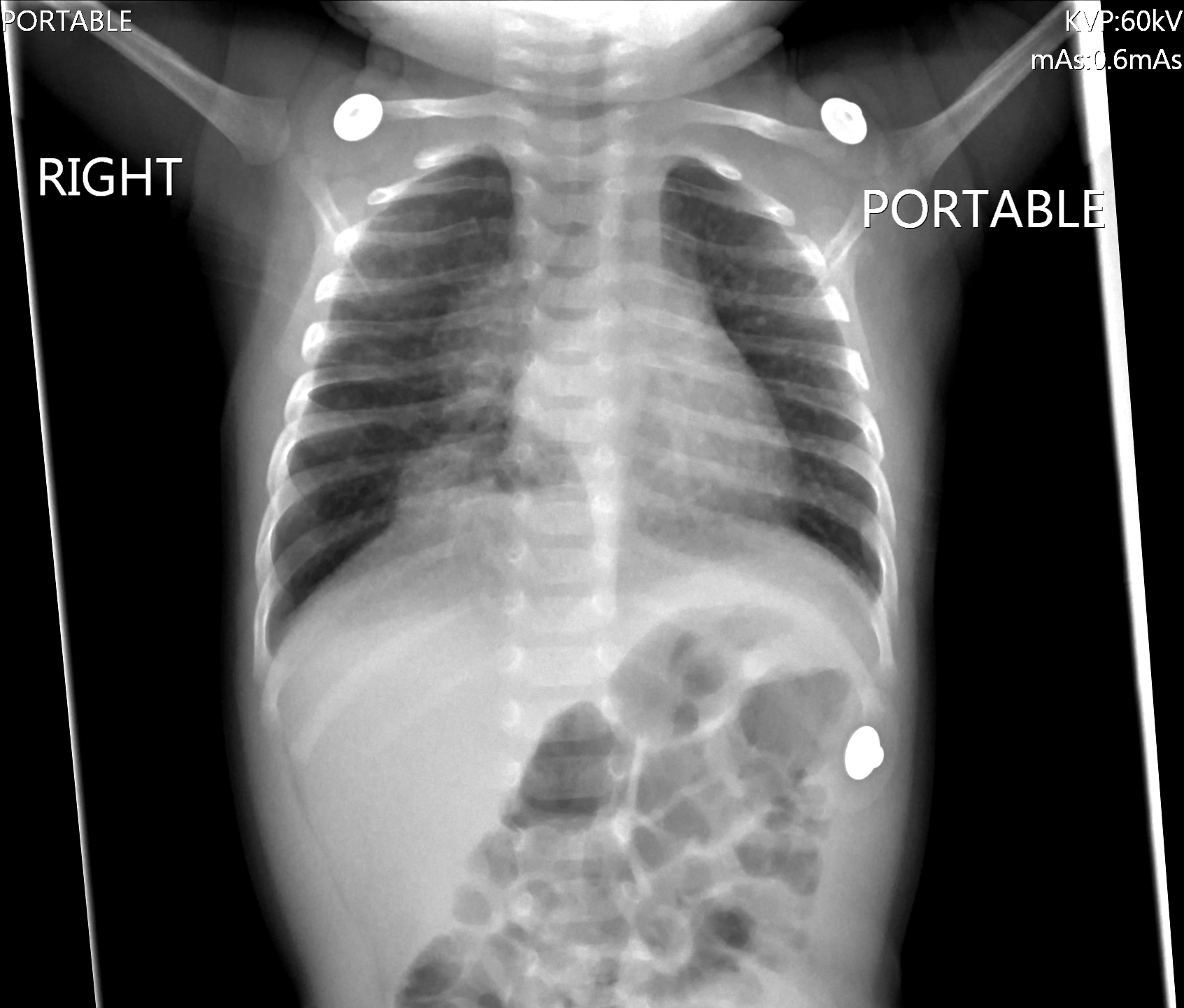

Supplement: Supplementary file 7 [file jetem-8-1-v18-supp7.jpg]

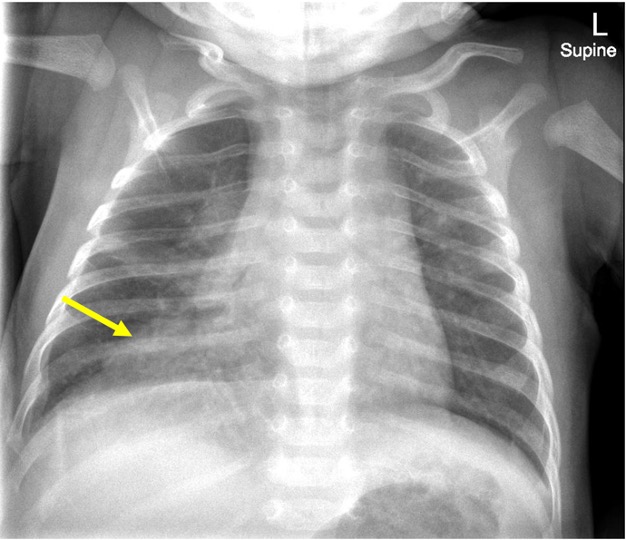

Supplement: Supplementary file 8 [file jetem-8-1-v18-supp8.jpg]

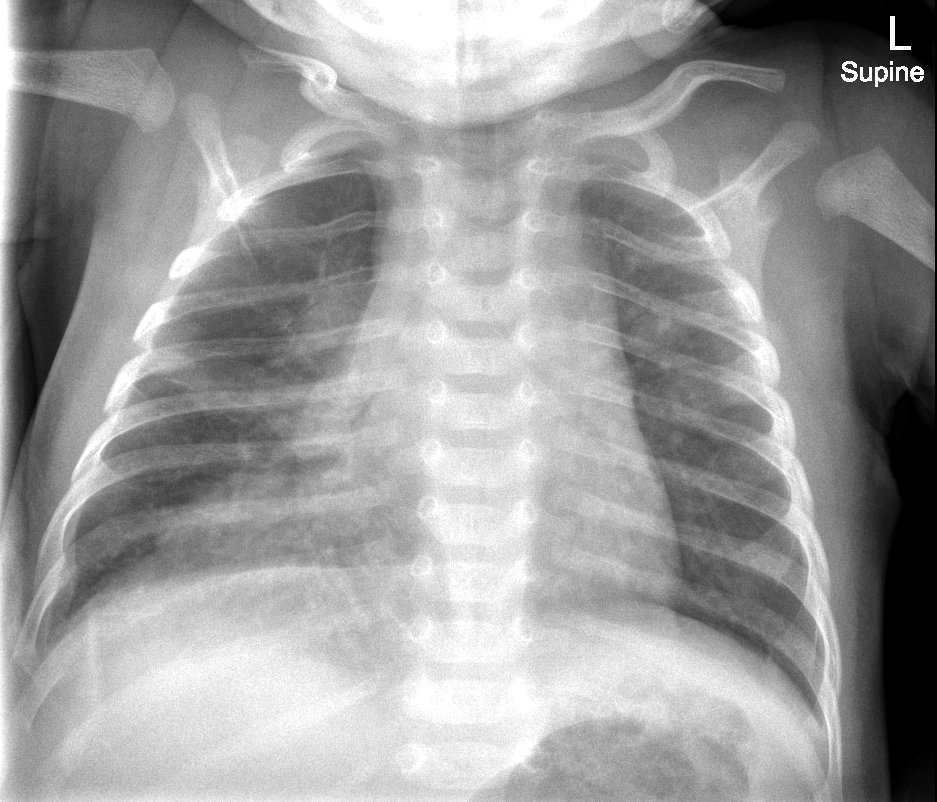

Supplement: Supplementary file 9 [file jetem-8-1-v18-supp9.jpg]

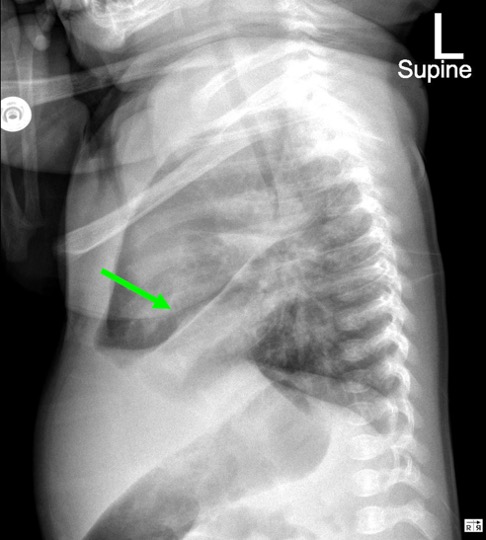

Supplement: Supplementary file 10 [file jetem-8-1-v18-supp10.jpg]

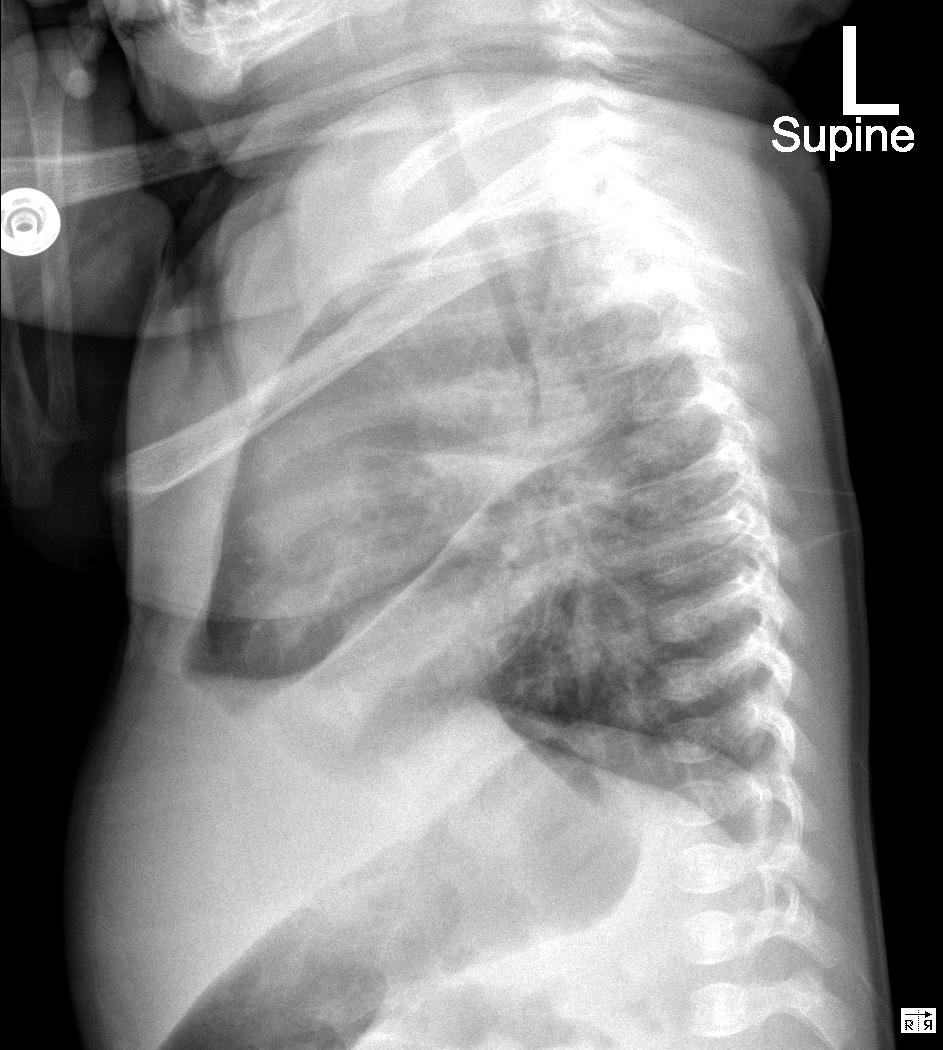

Supplement: Supplementary file 11 [file jetem-8-1-v18-supp11.jpg]

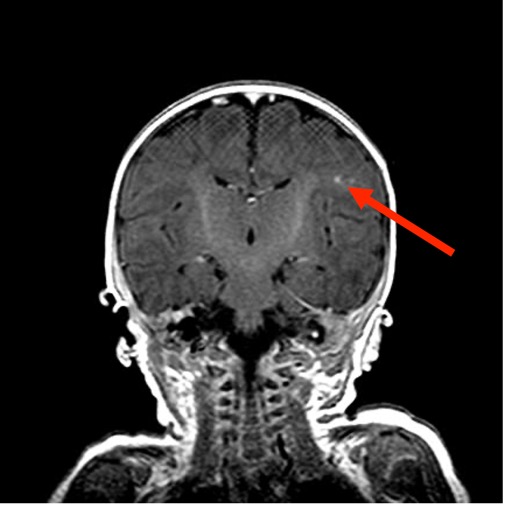

Supplement: Supplementary file 12 [file jetem-8-1-v18-supp12.jpg]

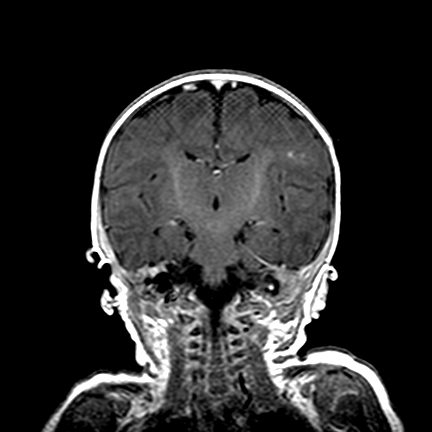

Supplement: Supplementary file 13 [file jetem-8-1-v18-supp13.jpg]
